# Unravelling the Sequential Interplay of Mutational Mechanisms during Clonal Evolution in Relapsed Pediatric Acute Lymphoblastic Leukemia

**DOI:** 10.3390/genes12020214

**Published:** 2021-02-02

**Authors:** Željko Antić, Stefan H. Lelieveld, Cédric G. van der Ham, Edwin Sonneveld, Peter M. Hoogerbrugge, Roland P. Kuiper

**Affiliations:** 1 Princess Máxima Center for Pediatric Oncology, 3584 CS Utrecht, The Netherlands; antic.zeljko@mh-hannover.de (Ž.A.); s.h.lelieveld@prinsesmaximacentrum.nl (S.H.L.); c.g.vanderham-2@prinsesmaximacentrum.nl (C.G.v.d.H.); e.sonneveld-2@prinsesmaximacentrum.nl (E.S.); p.m.hoogerbrugge@prinsesmaximacentrum.nl (P.M.H.); 2 Dutch Childhood Oncology Group, 3584 CS Utrecht, The Netherlands; 3 Department of Genetics, University Medical Center Utrecht, 3508 AB Utrecht, The Netherlands

**Keywords:** pediatric acute lymphoblastic leukemia, mutational signatures, clonal evolution, relapse development, APOBEC activity, thiopurine-associated DNA damage

## Abstract

Pediatric acute lymphoblastic leukemia (ALL) is the most common pediatric malignancy and is characterized by clonal heterogeneity. Genomic mutations can increase proliferative potential of leukemic cells and cause treatment resistance. However, mechanisms driving mutagenesis and clonal diversification in ALL are not fully understood. In this proof of principle study, we performed whole genome sequencing of two cases with multiple relapses in order to investigate whether groups of mutations separated in time show distinct mutational signatures. Based on mutation allele frequencies at diagnosis and subsequent relapses, we clustered mutations into groups and performed cluster-specific mutational profile analysis and de novo signature extraction. In patient 1, who experienced two relapses, the analysis unraveled a continuous interplay of aberrant activation induced cytidine deaminase (AID)/apolipoprotein B editing complex (APOBEC) activity. The associated signatures SBS2 and SBS13 were present already at diagnosis, and although emerging mutations were lost in later relapses, the process remained active throughout disease evolution. Patient 2 had three relapses. We identified episodic mutational processes at diagnosis and first relapse leading to mutations resembling ultraviolet light-driven DNA damage, and thiopurine-associated damage at first relapse. In conclusion, our data shows that investigation of mutational processes in clusters separated in time may aid in understanding the mutational mechanisms and discovery of underlying causes.

## 1. Introduction

Pediatric acute lymphoblastic leukemia (ALL) represents the most common pediatric malignancy [1,2,3,4]. Despite improvements in treatment, around 10–15% of the children do not achieve long-term remission and outcome among them remains poor [5,6]. Previous next-generation sequencing (NGS) studies revealed unprecedented diversity in the genomic alteration of ALL [7,8,9,10,11,12]. However, only a small subset of these alterations occurs in known cancer-driver genes, which have the potential to initiate and propel disease progression. Furthermore, these studies revealed genetic alterations which are not essential for cancer development but may drive treatment resistance and eventually give rise to a relapse [7,8,13,14,15,16,17,18,19,20]. Although the majority of genetic alterations in ALL represent passenger mutations and do not confer with selective advantage of leukemic cells, they still contain valuable information about tumor evolution, clonal dynamics, and mechanisms driving mutagenesis [7,8,11,12,21,22,23,24].

In contrast to adult cancers, where external factors like ultraviolet (UV) light exposure, tobacco smoke, and alcohol consumption contribute to the incidence of cancer in the population, pediatric cancers are more likely the result of dysregulated intrinsic processes which directly impact normal development [8,11,12,21,23,24,25]. In some cases, a mutational process can become active in a (pre)malignant cell that may accelerate mutation accumulation and disease progression. Examples of these mutational processes are aberrant activity of the activation induced cytidine deaminase (AID)/apolipoprotein B editing complex (APOBEC) class cytidine deaminases and mismatch repair deficiency (MMR), processes that also have been reported in ALL [8,11,12,21,23,24,26]. Each of these mutagenic processes shows biases represented by specific changes which occur in distinct genomic contexts [8,21,23]. These changes are recognized as footprints of underlying biological processes, also known as mutational signatures [21,23,24]. For example, mutations driven by aberrant AID/APOBEC activity typically present as C > T/G substitutions in TpCpN context. In addition, certain mutational processes may be accompanied by additional features, e.g., small indels in simple repeats in MMR deficiency, while others exhibit stronger mutational patterns in specific genomic regions, e.g., introns in AID/APOBEC-driven mutational signature [21,23,24]. So far, 72 single-base substitution (SBS) signatures have been described in the Catalogue of Somatic Mutations in Cancer (COSMIC), many of which are of unknown etiology [21]. Tumor samples taken at defined disease stages may reveal the footprints of several of these processes, but these mutational processes may have been active at different times and in different cells during tumor development. For example, compared to tumors at primary diagnosis, relapses may exhibit specific mutational signatures that are a direct consequence of prior treatment with, e.g., DNA damaging agents as part of multidrug chemotherapy regimen [23,27]. Unraveling mutational patterns of processes driving mutagenesis in a spatial and temporal manner may give insights into the intrinsic and external influences and impact of therapy on disease evolution. 

We and others have previously demonstrated that whole genome sequencing (WGS) of leukemia at diagnosis and relapse may aid in deciphering unique evolutionary trajectories of individual clones emerging from each other due to different quantities in which these clones occur at each time point [7,8,22,27]. This provides the opportunity to study mutational mechanisms that, for example, occurred before and after therapy, or can even be taken a step further by sequencing tumors from multiple tumor sites. Sequencing of multiple samples with different spatio-temporal origin can aid in discriminating clusters of mutations with similar clonal dynamics, which opens the possibility to allocate mutational mechanisms to individual clones with different behavior in time and space. 

**Figure 1:**
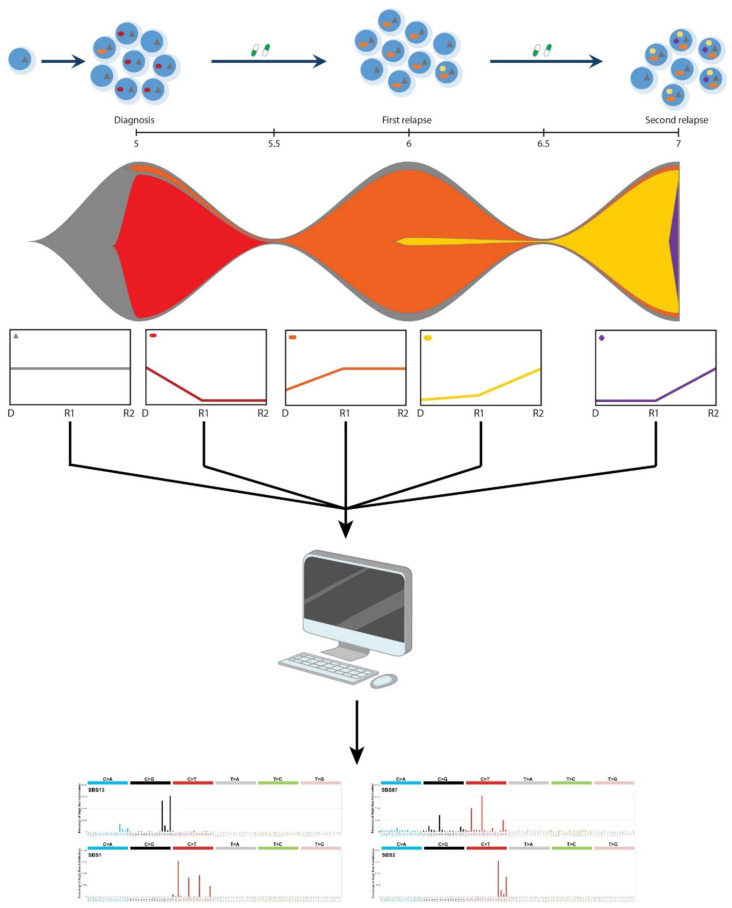
Schematic representation of the approach. Mutational profiles are analyzed in clusters of mutations that follow similar dynamics over different time points. Leukemic cells are thought to collect mutations sequentially, indicated as different colored symbols in each cell (top panel). New clones evolving from the ancestral clone, depicted in different colors in the fish plot, can be extracted based on the dynamics of the variant allele frequency (VAF) between different time points, which are then used to investigate active mutational mechanisms by analyzing their mutational profiles and performing de novo signature extraction in a clone-specific manner.

Based on our previous findings, we hypothesize that analyzing clusters of mutations in a clone-specific manner will improve the identification of mutational processes that are active in individual leukemic clones and different stages of disease progression (Figure 1). Here, we show a proof-of-principle for this approach by performing whole genome sequencing of 10 samples from two pediatric ALL patients with multiple relapses, revealing the sequential action of multiple mutational mechanisms in each of the two cases.

## 2. Materials and Methods

### 2.1. Patient Samples

In order to follow dynamics of a maximal number of individual somatic mutations at multiple time points during leukemia progression, we selected two patients with multiple relapses and performed WGS. Individual clones within a leukemia may have different dynamics during disease progression, which can be disentangled with greater resolution when more time points are available.

**Figure 2:**
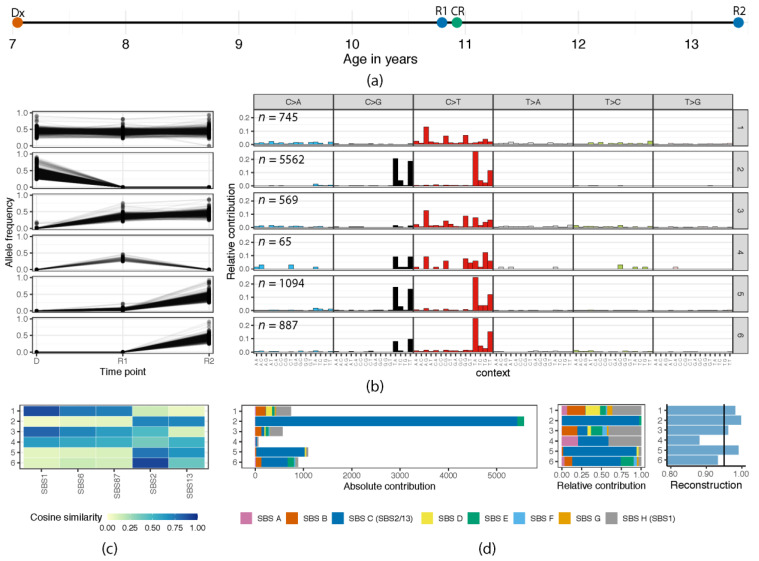
Analysis of mutational clusters in patient 1. (**a**) Schematic timeline of the analyzed samples with age at diagnosis (Dx) or relapse (R). A complete remission sample (CR) after first relapse was used as a normal control. (**b**) Clustering of high-confident somatic single-base substitutions based on allele frequencies at time of diagnosis and the two relapses (**left**) and the associated 96 trinucleotide mutation profiles (**right**). The clusters represent a (pre)leukemic ancestral clone (cluster 1), falling (cluster 2) and two rising clones (clusters 3 and 4) at first relapse, and rising clones at second relapse (clusters 5 and 6). The number of somatic mutations per cluster is indicated in the top right corner of each panel. (**c**) Heatmap showing the COSMIC signatures with a cosine similarity higher than 0.7 to the mutational profiles of at least one of the six clusters. For the complete overview of the six clusters to all 72 COSMIC v3 signatures, see Supplementary Figure S3. (**d**) The absolute and relative contribution and reconstruction of eight extracted de novo signatures (Supplementary Figure S4) for each of the clusters. The right panel depicts cosine similarities between the actual mutational profiles and the reconstructed profile based on the identified de novo extracted signatures, with a reliability threshold set at 90%.

Patient 1 was a girl with Down syndrome who developed *CRLF2*-positive B-cell leukemia at the age of 7, who received treatment according to the Dutch Childhood Oncology Group (DCOG) ALL9 non-high-risk protocol. After achieving complete remission, the patient experienced a first relapse 4 years after initial diagnosis and a second relapse 6.9 years after diagnosis (Figure 2A). She achieved complete remission and received an allogeneic stem cell transplant (SCT) but relapsed one year later, from which she passed away. No material was available from the third relapse. 

**Figure 3:**
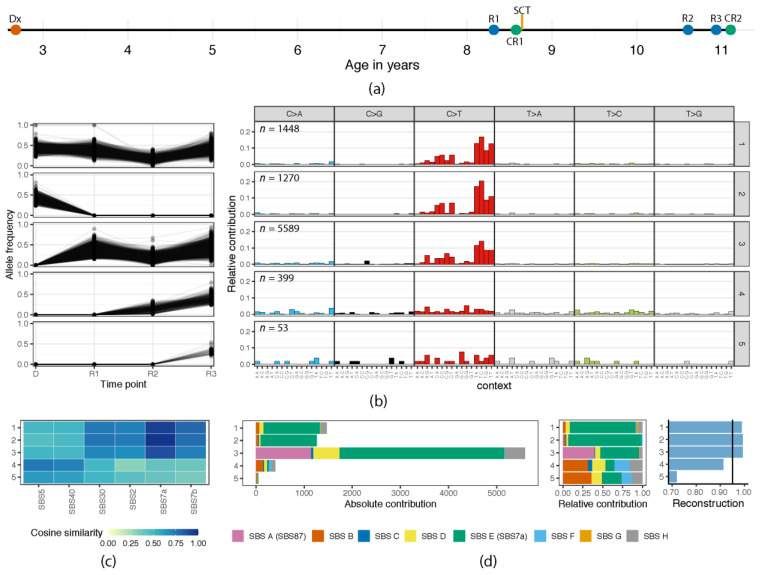
Analysis of mutational clusters in patient 2. (**a**) Schematic timeline of the analyzed samples with the age at diagnosis (Dx) or relapse (R). The patient received an allogenic stem cell transplantation (SCT) after his first relapse. Complete remission before (CR1) and after (CR2) were sequenced to correct for patient- and donor-derived normal variation. (**b**) Clustering of all high-confidence somatic single-base substitutions based on allele frequencies at diagnosis and three relapses (**left**) and the associated 96-trinucleotide mutation profiles corresponding to these clusters (**right**). The clusters represent a (pre)leukemic ancestral clone (cluster 1), falling (cluster 2) and rising clone (cluster 3) at relapse 1, and rising clones at relapse 2 (cluster 4) and relapse 3 (cluster 5). Total number of somatic mutations in each cluster is indicated in the top right corner of each profile. (**c**) Heatmap showing the COSMIC signatures with a cosine similarity higher than 0.7 to the mutational profiles of at least one of the five clusters. For the complete overview of all five clusters to the 72 COSMIC signatures, see Supplementary Figure S3. (**d**) The absolute and relative contribution of eight extracted de novo signatures (Supplementary Figure S4) for each of the clusters. The contribution of SBSE (with a cosine similarity of 0.98 to COSMIC signature SBS7a) in clusters 1, 2, and 3 is 93%, 97%, and 89%, respectively. The contribution of SBSA (with a cosine similarity of 0.97 to COSMIC signature SBS87) in cluster 3 is estimated to be 39%. The right panel depicts cosine similarities between the actual mutational profiles and the reconstructed profile based on the identified de novo extracted signatures, with a reliability threshold set at 90%.

Patient 2 is a boy who developed type B-cell leukemia at the age of 2 and was treated according to the DCOG-ALL10 standard risk treatment protocol. A preserved *DDX3X-MLLT10* gene fusion was detected at all time points, but no subtype-specific abnormalities were identified. First relapse occurred 5.7 years after initial diagnosis, followed by a second relapse at 7.9 years and a third relapse at 8.3 years after initial diagnosis. As part of the treatment of the first relapse, this patient received an allogenic SCT (Figure 3A). 

DNA was isolated from mononuclear cells derived from bone marrow or peripheral blood. The percentage of blast cells in tumor samples was high for most of the samples (>80% for 6 samples) (Supplementary Table S1). In accordance with the Declaration of Helsinki, informed written consent was obtained from all patients and/or their legal guardians before enrolment in the study and the DCOG institutional review board approved the use of excess diagnostic material for this study (PMCLAB2019.054).

### 2.2. Whole Genome Sequencing

Whole genome sequencing for patient 1 and patient 2 was performed at Novagene (Hong Kong, China) and the Hartwig Medical foundation (Amsterdam, The Netherlands), respectively. The library was constructed using NEBNext DNA Library Preparation Kit, following sequencing on an Illumina NovaSeq 6000 platform using 150 base-pair paired-end reads. The sample-specific overview of achieved sequencing depth can be found in Supplementary Table S1. All samples were aligned to the HG38/GRCh38 of the human reference genome by using the burrow-wheelers aligner (BWA) [28]. Duplicate reads were marked using Picard. Tumor purity estimations were performed based on the allele frequencies of high-quality somatic variants detected in the WGS data. Manta (version 1.6.0) was used for structural variant detection on the WGS data [29].

### 2.3. Somatic Variant Calling, Annotation, and Filtering

Somatic variants were called by the MUTECT2 software of GATK package version 4.1.1.0 followed by the FilterMutectCalls function as recommended by the authors. We removed the somatic variant that did not have a “PASS” filter status to ensure the highest likelihood of true somatic variants. Next, we annotated the filtered somatic variants with the Variant Effector Prediction (VEP) version 92 [30]. Somatic variants were annotated with: (i) Frequencies of the 76,156 whole genome sequenced samples from the GnomAD release 3.0 [31], and (ii) frequencies from 498 whole genome sequenced samples of the Genome of the Netherlands [31,32].

Somatic variants were filtered on the following criteria: (i) Allele Frequency in the GnomAD database and the Genome of the Netherlands below 0.01, (ii) a minimal overall coverage of at least 20X in all samples and a minimum of at least 3 reads containing the variant, (iii) a minimal variant frequency of 25% in at least one of the samples from a patient, (iv) location outside the centromere locations (as defined in the UCSC genome browser), and (v) no variant reads in the control samples. 

### 2.4. Mutation Clustering

We applied K-mean clustering, integrated in R package stats (version 4.0.2), of high-confidence somatic variants to define clusters of variants with distinctive patterns of variant allele frequency (VAF) change between time points. These distinct clusters represent the same evolutionary trajectories as the cells from which they originate and, therefore, these clusters can be used to track dynamics of individual clones. For Patients 1 and 2, we created 10 and 15 clusters, respectively (Supplementary Figures S1 and S2). Next, to increase the number of mutations per cluster, we merged clusters with the same evolutionary trajectory together based on manual inspection. Finally, we cleaned the merged clusters of outlier mutations and divided the cleaned clusters to create biological relevant clusters that we subjected to mutational signature analysis (Supplementary Figures S1 and S2).

### 2.5. Mutational Profile Analysis

For each cluster of somatic substitutions, the 96-trinucleotide count matrices and mutation profiles were computed and visualized by the R package MutationalPatterns (version 2.0.0) [33]. For each cluster of mutations, we computed the cosine similarity of the 96-trinucleotide profile to the repertoire of 72 known single-base substitution (SBS) signatures reported in the Catalogue of Somatic Mutations in Cancer (COSMIC v3, URL: https://cancer.sanger.ac.uk/cosmic/signatures/SBS/index.tt). The cosine similarity is a measure ranging from 0 to 1 and is used to compute differences between two mutational profiles, where a value of 1 indicates an identical profile. Cosine similarity scores between the profiles of the clusters and the COSMIC signatures aids in the identification of recurrent active mutational mechanisms. 

### 2.6. De Novo Mutational Signature Extraction

Mutational signatures were de novo extracted from the 96-trinucleotide mutation count matrix using non-negative matrix factorization (NMF). We used the R packages MutationalPatterns (version 2.0.0) [33] and NMF (version 0.23.0) [34] to perform de novo signature extraction on the profiles of the somatic mutation clusters. To increase the power to perform de novo signature extraction, somatic variants of 214 whole genome sequenced pediatric ALL patients from a recent pan-cancer study were included [11]. The annotation and filtering of these somatic mutations were performed with the same pipelines and settings as the seven in-house sequenced tumor samples. We combined the 96-trinucleotide count matrices of the seven tumors of our two patients and the 214 and performed the de novo extraction of signatures on the combined set of 221 samples. The relative and absolute contribution of the de novo extracted signatures in the mutational profiles of the somatic mutation clusters were computed using the MutationalPatterns R package. Reconstruction of mutational profiles using de novo extracted mutational signatures was performed using the MutationalPatterns package in R. 3. 

## 3. Results

In order to unravel somatic mutations at each time point during tumor evolution, we performed WGS of tumor samples at diagnosis, remission, and all subsequent relapses of two patients diagnosed with B-cell precursor acute lymphoblastic leukemia (BCP-ALL). We specifically selected cases with multiple relapses, because they offer much greater resolution to distinguish individual clones, compared to non-relapsed cases. Since patient 2 received an allogeneic SCT before his second relapse, we also sequenced a remission sample taken after SCT in order to filter out donor-derived variants identified in the second and third relapse. In total, we detected 8922 and 8759 single-base substitutions and 686 and 646 indels in patients 1 and 2, respectively (Supplementary Tables S2–S4). Since the association between mutational signatures and underlying processes is best understood for single-base substitutions [21], we focused on this type of mutations in the present study.

Using K-mean clustering of the mutation allele frequencies observed at each time point, we extracted multiple clusters of single-base substitutions that followed similar dynamics over the different time points for both patients. These clusters were subsequently manually curated based on biological criteria, resulting in six and five clusters of sufficient size or biological relevance for patients 1 and 2, respectively (Supplementary Figures S1 and S2). Interestingly, the mutational profiles of the defined clusters in each of the two patients were highly different, even between clusters that co-occurred at one or more time points (Supplementary Table S5). Furthermore, the mutation profiles of some of these clusters showed high similarity with known COSMIC signatures (Supplementary Table S6), suggesting that indeed clone-specific mutational processes could be revealed in this manner. Below, we will describe the clonal trajectories with underlying mutational processes in more detail. 

### 3.1. (Re)Activation of Aberrant AID/APOBEC Expression Follows Evolution of Individual Clones

In patient 1, we identified a total of six clusters of somatic single-base substitutions that presented with different dynamics between diagnosis, first relapse, and second relapse (Figure 2B, Supplementary Figure S1). Cluster 1 was composed of 745 mutations that were preserved between all time-points, suggesting their origin from a (pre)leukemic ancestral clone. In addition, we observed a large second cluster of mutations (5562 mutations) representing a falling clone that was dominant at diagnosis but disappeared at first relapse. The mutations in clusters 3 and 4 appeared at time of first relapse, but whereas the mutations in cluster 3 (*n =* 562) were preserved in the second relapse, the cluster 4 mutations (*n =* 65) were lost at relapse 2 (R2). This suggests that the dominant clone in the first relapse contained mutations from both of these clusters but was eradicated during treatment. In contrast, a minor preceding clone, which did not carry the cluster 4 mutations, evolved into a second relapse. This second relapse also carried new mutations, of which about half (cluster 5; *n =* 1094) were already detectable in low amounts in relapse 1, whereas 887 mutations (cluster 6) appeared to be newly acquired. Therefore, the six mutational clusters represent distinct mutational episodes during progression of the disease in this patient.

Next, we examined the mutational profiles of each of these clusters, which revealed that four of them showed high similarity with known COSMIC signatures (Figure 2C and Supplementary Tables S5 and S6). Cluster 1, which carried the preserved ancestral mutations, showed high similarity with the clock-like mutational signature SBS1 (cosine similarity = 0.89). These mutations are associated with spontaneous deamination of methylated cytosines at CpGs and may have occurred during the pre-malignant phase of the leukemia-initiating cell. Three clusters showed high similarity with the COSMIC signatures SBS2 and SBS13, which are both attributed to AID/APOBEC mutagenesis. Cosine similarity of the merged SBS2/13 signature with clusters 2, 5, and 6 was 0.98, 0.99, and 0.95, respectively (Supplementary Figure S3). Clusters 3 and 4 appeared as mixed signatures, which were likely at least in part composed of SBS1 and SBS2/13. To confirm that this was indeed the case, we performed a de novo signature extraction in which we included all individual clusters as well as 214 publicly available ALL samples (see Methods, Supplementary Tables S7 and S8). The de novo signature analysis revealed eight signatures with high cosine similarities ranging to COSMIC signatures that resembled a variety of mutational processes (Supplementary Figure S4, Supplementary Tables S7–S9). Four extracted signatures were prominently found in at least one of the clusters from the two patients (Figure 2D and Figure 3D). In patient 1, this includes SBSH (cosine similarity of 0.98 to COSMIC signature SBS1), which represents 41% of the mutations in both clusters 1 and 3 (Figure 2D). Signature SBSC (cosine similarity of 0.98 to the combined COSMIC signatures SBS2 and SBS13), which is indeed the prominent signature in clusters 2, 5, and 6, contributed 92%, 97%, and 61% of all somatic substitutions (Figure 2D). Also, in cluster 4, which only contains 65 mutations, resulting in lower accuracy, AID/APOBEC activity appears to be responsible for 38% of the mutations. Furthermore, we noted that clusters 5 and 6 differ in their relative contributions between SBS2 and SBS13 (cluster 5: 0.75 and 0.65 vs. cluster 6: 0.91 and 0.40, respectively; Figure 2C), suggesting that SBS13 mutations were more prominent in the cluster 5 mutations, which were already present subclonally at R1 compared to the cluster 6 mutations. In conclusion, the six clusters of mutations demonstrate that AID/APOBEC-mediated mutagenesis appeared to be active in emerging clones at different stages of leukemia development in patient 1, particularly at diagnosis and relapse 2. Strikingly, these SBS2/13 mutations were hardly preserved but the underlying cause remained present in the different stages of disease.

### 3.2. Episodic Activity of Mutational Mechanisms

In patient 2, we identified a cluster of preserved mutations (cluster 1), a falling clone at diagnosis (cluster 2), and rising clones at the first, second, and third relapse (clusters 3, 4, and 5; Figure 3B and Supplementary Figure S2). Therefore, similar to patient 1, patient 2 presented with different dominant clones at diagnosis and first relapse, which shared the cluster 1 mutations combined with the mutations in either cluster 2 or cluster 3, respectively. Strikingly, however, clusters 1, 2, and 3 were highly similar in their mutational profile and strongly resembled a known COSMIC mutational signature associated with UV-associated DNA damage (SBS7a, cosine similarities of clusters 1, 2, and 3 were 0.93, 0.97, and 0.89, respectively; Figure 3C and Supplementary Table S6). Thus, this mutational mechanism strongly contributed to the majority of mutations in both diagnosis and first relapse. The mutations in clusters 4 and 5 were clearly different, suggesting that after first relapse, this mutational mechanism was no longer active. To gain more insight into the mutational mechanisms active at all time points, de novo signature extraction on the individual clusters was performed, which confirmed the presence of the mutational signature SBS7a, in clusters 1, 2, and 3 (Figure 3C, Supplementary Tables S7 and S8). Signature SBS7a has been associated with UV light exposure and is commonly found in head and neck cancers and melanoma. Previous studies reported this mutational signature also in several pediatric ALL patients [11,27], but the etiology is still unknown. However, despite the fact that the number of mutations in clusters 4 and 5 was too low to reliably assign signatures, SBS7a seemed to be absent, suggesting that this mutational mechanism was no longer present after first relapse. Furthermore, SBS7a represented only 89% of the mutations acquired in relapse 1-specific cluster 3, and it may be possible that these mutations were already present at low levels at time of diagnosis. Additionally, relapse 1-specific cluster 3 showed a strong presence of a different, therapy-related signature, as 39% of the mutations in cluster 3 were assigned to de novo extracted signature SBSA that have a cosine similarity of 0.97 to COSMIC signature SBS87. SBS87 was recently identified in relapsed BCP-ALL and was associated with thiopurine treatment [27]. The latter study reported pathogenic mutations in the cytosolic 5’-nucleotidase II gene *NT5C2* in five patients with SBS87-associated mutations at relapse, but this mutation was not identified in patient 2, and no other pathogenic mutations were found that could explain the presence of thiopurine-related damage (Supplementary Tables S8 and S9).

## 4. Discussion

Pediatric ALL is a heterogeneous disease characterized by the presence of multiple leukemic clones [7,8,9]. Clonal diversification at different stages during disease development may be a ramification of mutational mechanisms that are active during episodes of disease progression or throughout the entire disease course [8,26,27]. These processes drive clonal heterogeneity and may lead to selection of therapy-resistant clones [27]. Various mutational mechanisms have been identified in (relapsed) ALL cases, but the spatial and temporal activity of these mechanisms is still far from understood. In this whole genome sequencing study on two BCP-ALL patients with multiple relapses, we demonstrated that by clustering mutations based on the clonal dynamics during disease progression, distinct mutational processes can be unraveled, and their timing can be specified. This proof-of-principle can be applied to past and future whole genome sequencing studies involving multiple samples of the same tumor, separated in time or space in order to unravel the sequential interplay of mutational mechanisms during cancer progression. 

Both cases revealed complex evolutionary patterns, with multiple clones rising and falling during disease progression. The clusters of mutations underlying these individual clones revealed distinct mutational mechanisms in these patients. In patient 1, the outgrowth of multiple relapses appeared to be derived from subclones that acquired new mutations by a sustained AID/APOBEC-driven mutational mechanism. This mechanism is responsible for high mutational burden in a subset of ALL [8,23,27], and can be recognized by the presence of two distinct signatures, SBS2 and SBS13. These signatures are thought to arise from the same mutational mechanism (cytosines to uracil deamination at TpC dinucleotides), but whereas SBS2 directly results from the replication of a U:A mismatch, SBS13 appears to be caused by error-prone polymerases that fill in the excised uracil [23]. SBS2 and SBS13 often co-occur in similar amounts, which explains why the two patterns can be recognized as a single mutational signature [8]. Interestingly, however, we observed a difference in the relative contribution of SBS13 between clusters 5 and 6 in patient 1. Since cluster 5 contains relapse 2 mutations that were already present subclonally at the first relapse, this observation may be suggestive of a relatively higher level of uracil excision in the subclonal stage, for example because of lower replication rates. Inclusion of more ALL cases with underlying AID/APOBEC mutagenesis in future studies may further clarify this aspect.

In patient 2, two mutational mechanisms were identified which, in contrast to patient 1, were active only temporarily. The mutations detected at diagnosis could be linked to mutational signature SBS7a, which has been associated with DNA damage caused by UV light exposure [11,21,23,24,27]. SBS7a-associated mutations have been observed occasionally in ALL [11,27], but the underlying cause of these mutations in the bone marrow is still unknown. Acquired mutations in the first relapse also contained a substantial number of SBS7a-associated mutations, but in the later relapses, this mutational mechanism was no longer active. In fact, we cannot rule out the possibility that relapse 1 emerged from a subclone that was already present at diagnosis, and that the SBS7a mutations all arose before first diagnosis. Whereas SBS7a appears to be caused by an intrinsic process, a substantial number of mutations in the first relapse could be linked to thiopurine treatment (SBS87) [27]. Presence of these thiopurine-related scars may indicate that this therapy was (partially) ineffective, which may have contributed to the development of relapse. Strikingly, however, also, this mutational mechanism was no longer present in later relapses, despite the use of thiopurines in the ALL-R3-based therapy he received after his first relapse. The absence of thiopurine scars may suggest that the later relapses were either completely resistant or completely sensitive to thiopurine-induced DNA damage.

## 5. Conclusions

In summary, we showed that the use of samples taken at multiple time points during tumor evolution may improve separation of distinct cell populations that evolve independently, thereby unravelling clone-specific mutational mechanisms. Clonal inference can be further improved by deep targeted resequencing of somatic mutations, as well as utilization of limited dilution xenograft models, as previously demonstrated [8,35]. Furthermore, we showed that independent examination of mutational clusters reveals distinct mutational profiles which correspond to previously reported mutational signatures. We therefore conclude that analyzing mutational signatures in the clusters of mutations, in a clone-specific manner, can improve our understanding of mutational processes occurring in a single parental cell.

## Data Availability

The data presented in this study have been deposited in the European Genome-phenome Archive (https://ega-archive.org); accession number EGAS00001005001.

## Supplementary Materials

The following are available online at https://www.mdpi.com/2073-4425/12/2/214/s1, Figure S1: Mutation clustering in patient 1. Figure S2: Mutation clustering in patient 2. Figure S3: Cosine similarity heatmap of the mutational profiles. Figure S4: De novo mutational signature extraction. Table S1: Coverage statistics of the whole genome sequenced samples for both patients. Table S2: Mutational burden, number of acquired mutations, and number of lost mutations at each time point in patient 1 and patient 2. Table S3: High-confidence somatic mutations of Patient 1. Table S4: High-confident somatic mutations of Patient 2. Table S5: 96-trinucleotide count matrices and mutation profiles of the clusters of patients 1 and 2. Table S6: Cosine similarity scores between mutational profiles of patients 1 and 2 and 72 COSMIC signatures. Table S7: Mutational profiles of the eight de novo extracted signatures. Table S8: Cosine similarity scores between de novo extracted signatures and 72 COSMIC signatures. Table S9: Overview of the de novo extracted signatures.
