# Supplementary material for: Unravelling the Sequential Interplay of Mutational Mechanisms during Clonal Evolution in Relapsed Pediatric Acute Lymphoblastic Leukemia
Source: Genes (Basel). 2021 Feb 2;12(2):214. doi: 10.3390/genes12020214 (PMC7913080; doi:10.3390/genes12020214)
Supplement: Supplementary file 1 [file genes-12-00214-s001.zip › Antic_Lelieveld_genes_supplements.docx]

**SUPPLEMENTARY MATERIALS**

**Unravelling the sequential interplay of mutational mechanisms during clonal evolution in relapsed pediatric acute lymphoblastic leukemia**

Željko Antić^1^, Stefan H. Lelieveld^1^, Cédric G. van der Ham^1^, Edwin Sonneveld^1,2^, Peter M. Hoogerbrugge^1,2^, Roland P. Kuiper^1,3^

1. Princess Máxima Center for Pediatric Oncology, Utrecht, The Netherlands
2. Dutch Childhood Oncology Group, Utrecht, The Netherlands
3. Department of Genetics, University Medical Center Utrecht, Utrecht.

Statement of equal authors’ contribution:

ŽA and SHL contributed equally to this work.

Correspondence:

Roland P. Kuiper

Heidelberglaan 25

3584CS Utrecht, The Netherlands

E-mail: [r.kuiper@prinsesmaximacentrum.nl](mailto:r.kuiper@prinsesmaximacentrum.nl)

**
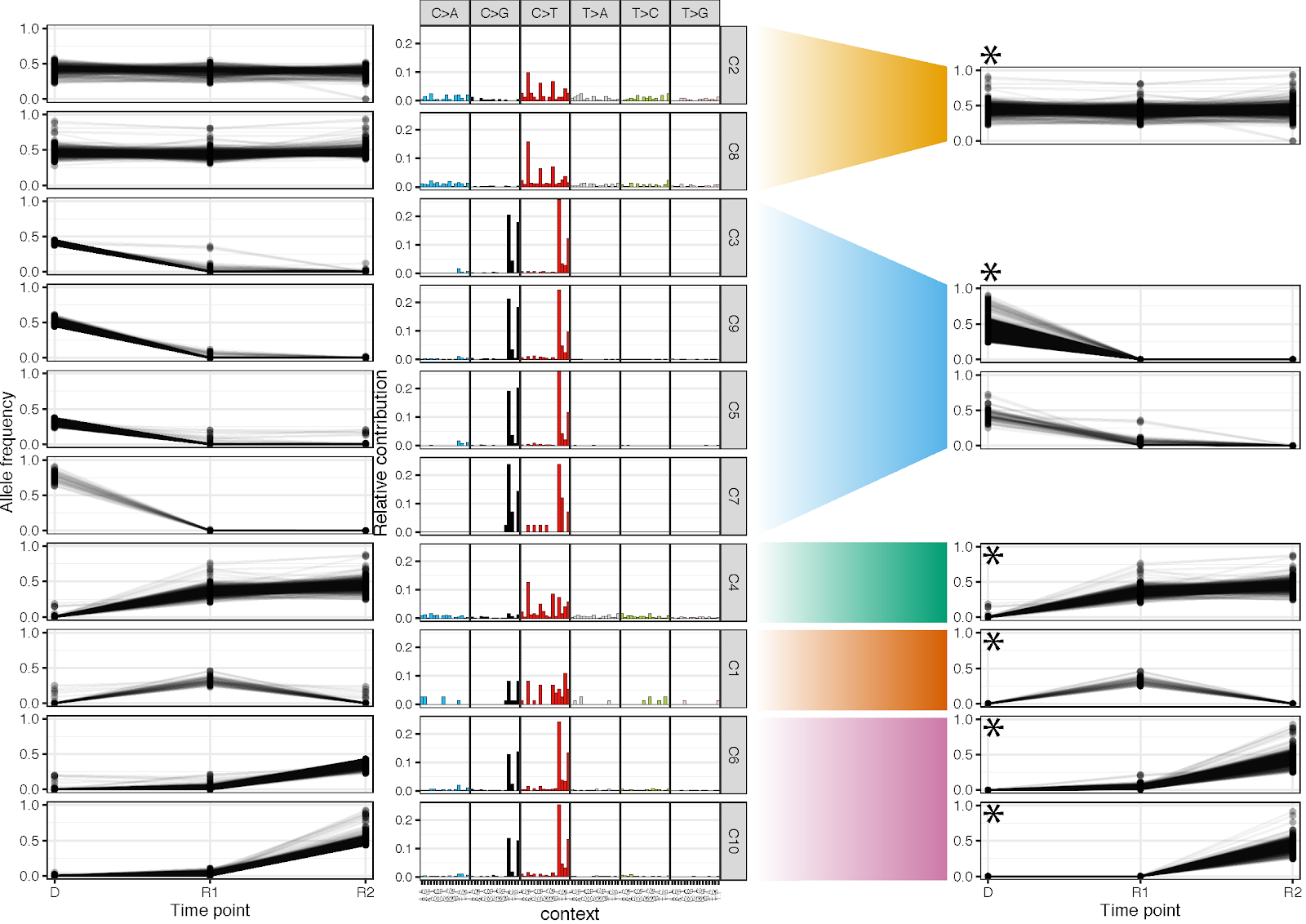
**

**Supplementary Figure 1** - Mutation clustering in patient 1. The left panel shows the 10 clusters (K-means clustering; K = 10) of high confident filtered somatic mutations identified in the whole-genome sequence experiments in patient 1. The middle panel shows the 96 trinucleotide mutation profiles corresponding to 10 clusters on the left. We manually merged clusters with the same evolutionary trajectory (left panel) and similar mutational profiles together. The clusters represent a (pre)leukemic ancestral clone (yellow), falling (blue) and two rising clones (green and red) at first relapse, and rising clones at second relapse (pink). Finally, we cleaned the merged clusters of outlier mutations and divided the cleaned clusters to create biological relevant clusters, indicated with an asterisk (*), that we subjected to mutational signature analysis.

*
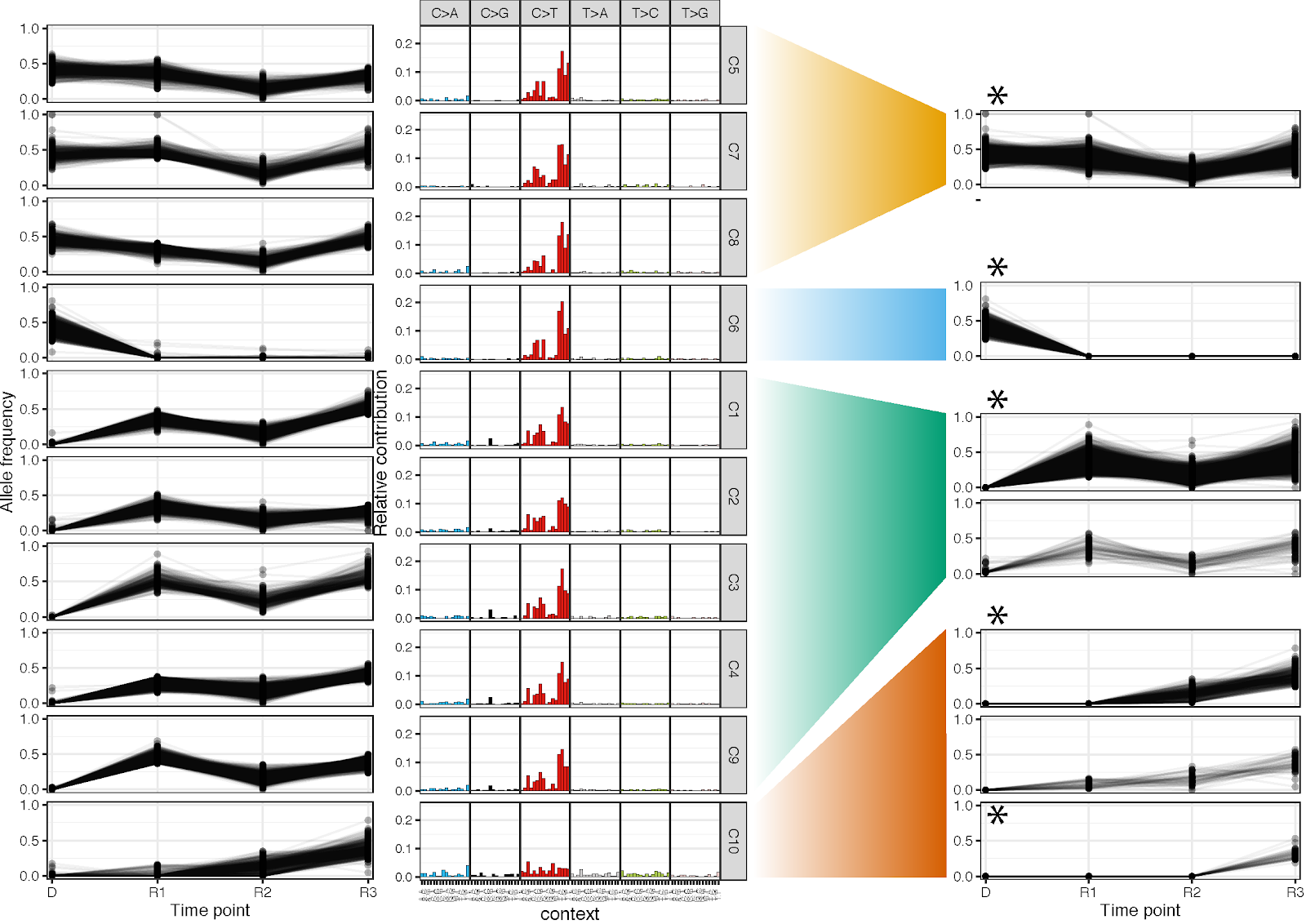
*

**Supplementary Figure 2** - Mutation clustering in patient 2. The left panel shows the 10 clusters (K-means clustering; K = 10) of high confident filtered somatic mutations identified in the whole-genome sequence experiments in patient 2. The middle panel shows the 96 trinucleotide mutation profiles corresponding to 10 clusters on the left. We manually merged clusters with the same evolutionary trajectory (left panel) and similar mutational profiles together. The clusters represent a (pre)leukemic ancestral clone (yellow), falling (blue) and rising clone (green) at relapse 1, and rising clones at relapse 2 (red; top cluster) and relapse 3 (red; bottom cluster). Finally, we cleaned the merged clusters of outlier mutations and divided the cleaned clusters to create biological relevant clusters, indicated with an asterisk (*), that we subjected to mutational signature analysis.

*
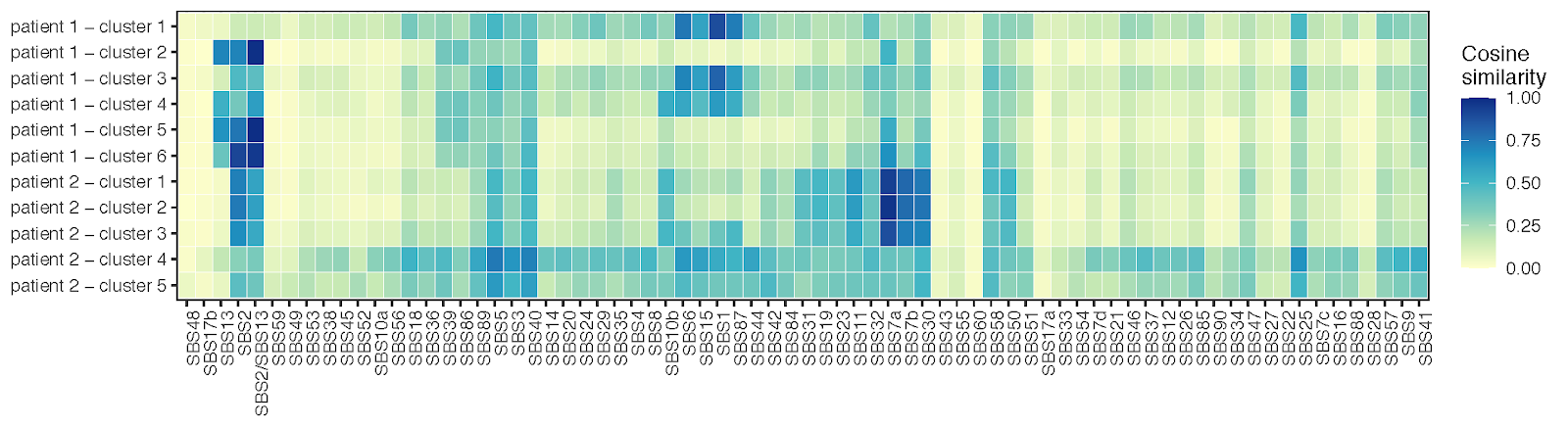
*

**Supplementary Figure 3** - Cosine similarity heatmap of the mutational profiles. Heatmap showing the cosine similarity of the profiles of the 6 clusters extracted from patient 1 and the 5 clusters extracted from patient 2 versus the 72 single base signatures present in the version 3.1 of the COSMIC database.

*
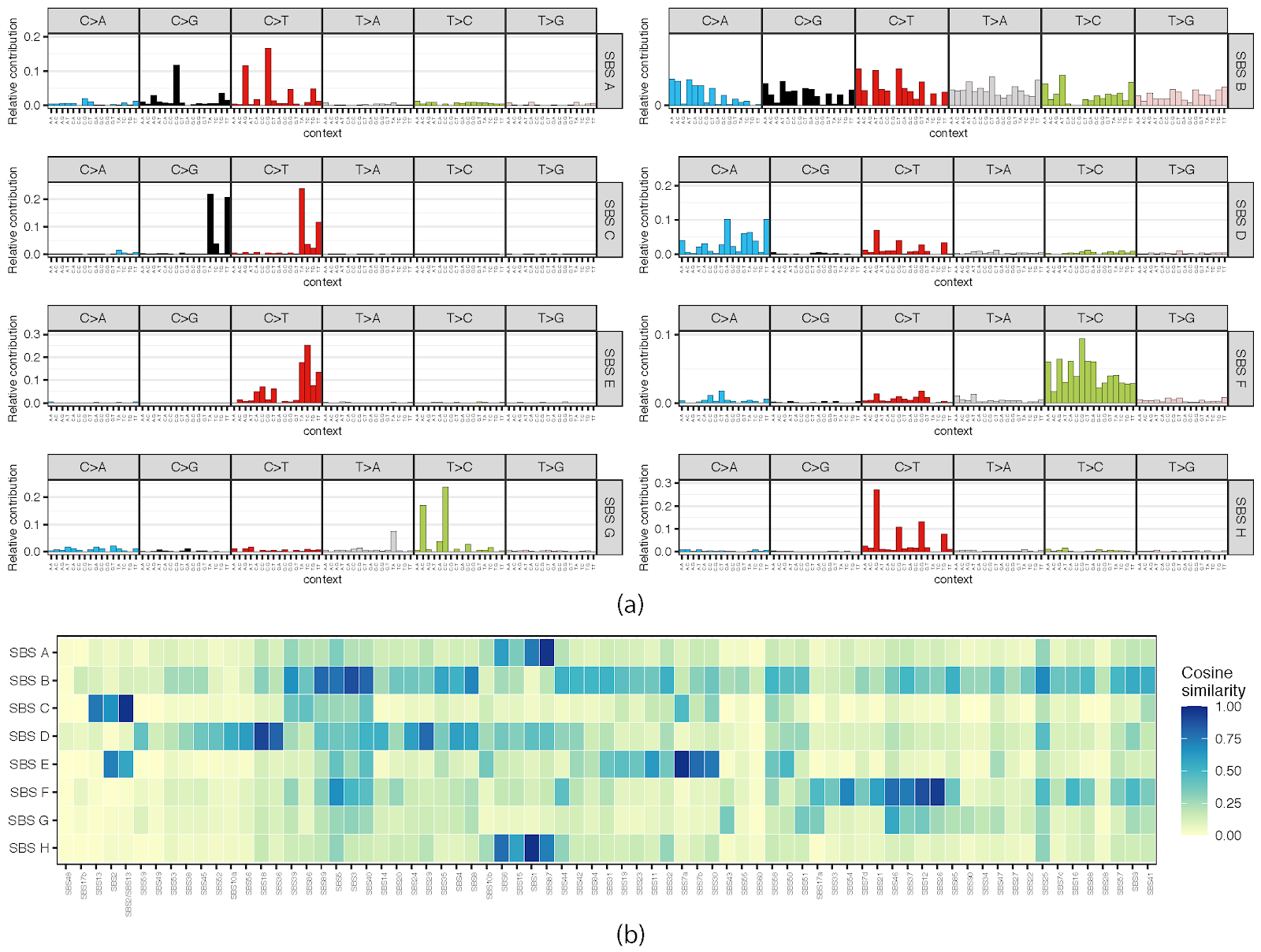
*

**Supplementary Figure 4** - *De novo* mutational signature extraction. (a) *De novo* mutational signature extraction yielded 8 single base substitution signatures that resemble known COSMIC mutational signatures. The four extracted signatures that were found to contribute significantly to the two patients in this study were SBS A (cosine similarity of 0.97 to COSMC signature SBS87), SBS C (cosine similarity of 0.98 to the merged COSMC signatures SBS2 and SBS13), SBS E (cosine similarity of 0.98 to COSMC signature SBS7a, and SBS H (cosine similarity of 0.98 to COSMC signature SBS1).The complete overview of the cosine similarity comparison to the COSMIC signatures can be found in Supplementary Tables 7, 8 and 9. (b) Cosine similarity heatmap of all eight extracted signatures to the known COSMIC signatures.

**Supplementary Table 1 -** Coverage statistics of the whole genome sequenced samples for both patients.

| **Patient** | **Sample** | | **Median depth** | **Percentage**  **≥20X coverage** | **Estimated**  **blast percentage** |
| --- | --- | --- | --- | --- | --- |
| 1 | Control 1 | CR1 | 34X | 96% |  |
| 1 | Diagnosis | Dx | 80X | 97% | ±94% |
| 1 | Relapse 1 | R1 | 83X | 97% | ±82% |
| 1 | Relapse 2 | R2 | 84X | 97% | ±86% |
| 2 | Control 1 | CR1 | 30X | 93% |  |
| 2 | Control 2 | CR2 | 46X | 97% |  |
| 2 | Diagnosis | Dx | 43X | 97% | ±88% |
| 2 | Relapse 1 | R1 | 36X | 96% | ±80% |
| 2 | Relapse 2 | R2 | 42X | 97% | ±34% |
| 2 | Relapse 3 | R3 | 37X | 96% | ±88% |

**Supplementary Table 2 -** Mutational burden, number of acquired mutations and number of lost mutations at each timepoint in patient 1 and patient 2

| **Patient** | **Timepoint** | **Mutational burden** | | |
| --- | --- | --- | --- | --- |
|  |  | **Total^1^** | **Acquired** | **Lost^2^** |
| 1 | Diagnosis | 6307 | 6307 | 5562 |
| 1 | Relapse 1 | 1379 | 634 | 65 |
| 1 | Relapse 2 | 3295 | 1981 | NA |
| 1 | Total (D+R1+R2) |  | 8922 |  |
| 2 | Diagnosis | 2718 | 2718 | 1270 |
| 2 | Relapse 1 | 7037 | 5589 | NA |
| 2 | Relapse 2 | 7436 | 399 | NA |
| 2 | Relapse 3 | 7489 | 53 | NA |
| 2 | Total (D+R1+R2) |  | 8759 |  |

^1^Total number of mutations present at that timepoint, ^2^Indicated are the mutations lost at the next timepoint

**Supplementary Table 9 -** Overview of the *de novo* extracted signatures.

| ***De novo* extracted SBS** | **COSMIC signature** | **Cosine similarity** | **Etiology** |
| --- | --- | --- | --- |
| SBSA | SBS87 | 0.97 | Thiopurine therapy related |
| SBSB | SBS3 | 0.88 | Defective homologous recombination-based DNA damage repair |
| SBSC | SBS3/13 | 0.98 | APOBEC 3A/3B |
| SBSD | SBS18 | 0.91 | Reactive Oxygen Species |
| SBSE | SBS7a | 0.98 | Exposure to UV-light associated |
| SBSF | SBS26 | 0.95 | MSI signatures |
| SBSG |  |  |  |
| SBSH | SBS1 | 0.98 | clock-like, age related |
